# Dynamic metabolic change of cancer cells induced by natural killer cells at the single-cell level studied by label-free mass cytometry†

**DOI:** 10.1039/d1sc06366a

**Published:** 2022-01-03

**Authors:** Zizheng Shen, Hansen Zhao, Huan Yao, Xingyu Pan, Jinlei Yang, Sichun Zhang, Guojun Han, Xinrong Zhang

**Affiliations:** Department of Chemistry, Tsinghua University Beijing 100084 China sczhang@mail.tsinghua.edu.cn; Institute of Medical Technology, Peking University Health Science Center Beijing 100191 China gjhan@bjmu.edu.cn; Peking University School and Hospital of Stomatology Beijing 100081 P. R. China; Department of Biomedical Engineering, Peking University Health Science Center Beijing 100191 P. R. China

## Abstract

Natural killer cells (NK cells) are important immune cells which have attracted increasing attention in cancer immunotherapy. Due to the heterogeneity of cells, individual cancer cells show different resistance to NK cytotoxicity, which has been revealed by flow cytometry. Here we used label-free mass cytometry (CyESI-MS) as a new tool to analyze the metabolites in Human Hepatocellular Carcinoma (HepG2) cells at the single-cell level after the interaction with different numbers of NK92 MI cells. A large amount of chemical information from individual HepG2 cells was obtained showing the process of cell apoptosis induced by NK cells. Nineteen metabolites which consecutively change during cell apoptosis were revealed by calculating their average relative intensity. Four metabolic pathways were impacted during cell apoptosis which hit 4 metabolites including glutathione (GSH), creatine, glutamic acid and taurine. We found that the HepG2 cells could be divided into two phenotypes after co-culturing with NK cells according to the bimodal distribution of concentration of these 4 metabolites. The correlation between metabolites and different apoptotic pathways in the early apoptosis cell group was established by the 4 metabolites at the single-cell level. This is a new idea of using single-cell specific metabolites to reveal the metabolic heterogeneity in cell apoptosis which would be a powerful means for evaluating the cytotoxicity of NK cells.

## Abstract 2

Label-free mass cytometry is utilized to study the dynamic metabolic change during apoptosis in HepG2 cells induced by NK92 MI cells at the single-cell level. The metabolic heterogeneity of individual HepG2 cells during apoptosis was revealed.

## Graphical abstract

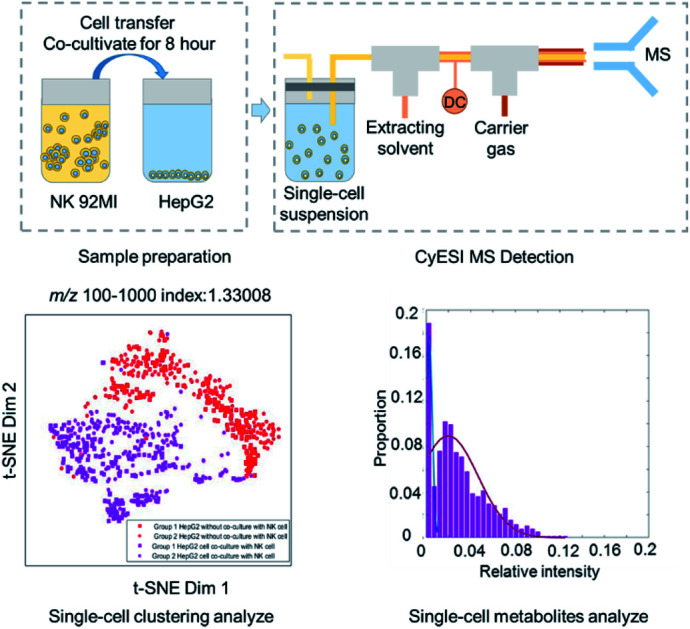

## Introduction

Natural killer cells (NK cells) are one of the most important immune cells in the human body, and are the initial immune component in the immune system to recognize and kill cancer cells.^1–4^ Different from B lymphocytes and T lymphocytes, the immune reaction mediated by NK cells can directly cause cytotoxicity without the introduction of antigens, so NK cells have more comprehensive ability in cancer cell killing.^5,6^ Up to now, the therapy of amplification and reinfusion of the heterogeneous NK cells has been successfully used to treat liver cancer,^7^ rectal cancer,^8^ breast cancer^9–11^ and acute myeloid leukemia^12–14^ in clinics. With the great improvement of NK cell therapy in recent years, the application of NK cells will be a more powerful weapon to fight against cancer cells.^15^

Although NK cells only account for 10–15% among the peripheral blood lymphocytes, they play an indispensable role in the immune process.^16,17^ Patients who lack NK cells or have decreased NK cells will show low efficiency when coping with cancer cells causing poor prognosis.^18^ Therefore, judging the cytotoxicity of NK cells has great significance in clinical immunotherapy and can be used to forecast the effectiveness of anti-cancer treatments. In the past few decades, the method for judging the cytotoxicity of NK cells by measuring released protein such as lactate dehydrogenase (LDH) was suggested. The principle of the LDH release method is detecting the amount of LDH released from the cancer cells killed by NK cells, and then calculating the proportion of dead cancer cells through a comparison with the control group.^19^ Because of the low cost-efficiency and reliable repeatability, the LDH release method has become the golden method in judging the cytotoxicity of NK cells.^20^ Limited by detection sensitivity, the LDH release method can only be used among population cells and provide the proportion of dead cells.^20^ Recently, flow cytometry (FCM)^21,22^ and mass cytometry (CyTOF)^23,24^ have been reported to distinguish the apoptotic state of cancer cells into early apoptosis, late apoptosis and dead cells at the single-cell level by measuring the apoptosis-associated cell membrane proteins.^25,26^ However FCM and CyTOF are inherently limited by the need for antibodies used to label the proteins.^27–29^

Compared with the detection of the released proteins or membrane proteins, the study of intracellular metabolites is of great value because metabolites are the end products of the life activities, which can reflect the changes of cell validity in a short time.^30–32^ The process of NK cells interacting with cancer cells also causes changes of intracellular metabolites.^1^ NK cells release pore-forming proteins (PFPs)^33–35^ and granzyme^36–38^ to form perforations on the cancer cell membrane and induce intracellular lysosome rupture, which eventually cause apoptosis. Intracellular metabolites can reflect and magnify the small changes in genes,^39^ mRNAs^40^ and proteins^41^ during the process of apoptosis induced by NK cells. Another inherent advantage is being label-free^42–44^ which can avoid the limitation of antibodies. Furthermore, intracellular metabolites contain a massive amount of data and can be used to detect and reveal potential biomarkers.^45–47^

In recent years, the analysis of intracellular metabolites based on population cells has been developed rapidly. High performance liquid chromatography (HPLC)^48^ is validly used to separate and identify metabolites from cancer cells. This method has been applied to study the principle of cell apoptosis and other life activities.^49,50^ HPLC needs a lot of time to separate the samples which is not conducive to rapid detection of cell metabolites.^48^ More importantly, the population cell metabolite data can only provide the average amount of metabolites which covers up the differences between individual cells during the cell apoptosis process induced by NK cells.^51,52^

Label-free mass cytometry (CyESI-MS)^53^ is a new tool developed by our group to satisfy the need for rapid analysis of metabolites at the single-cell level. In brief, the single-cell intracellular metabolites are extracted by the surrounding solvent in a capillary and ionized under a high-voltage electric field. CyESI-MS has been proved to provide a multitude of metabolite profiles at the single-cell level rapidly and conveniently, which make it possible to analyze the changes of intracellular metabolites during the cell apoptosis process. The single-cell metabolite profiles can also be applied to judge the activities of apoptotic pathways through further statistical analysis.

Herein we studied the dynamic metabolite changes of cancer cells induced by NK cells using CyESI-MS at the single-cell level. With the increasing ratio of NK cells (effector cells) and cancer cells (target cells), the metabolite fingerprint of cancer cells changed consecutively and 19 metabolites related to cell apoptosis such as glutathione (GSH), creatine, glutamic acid and taurine also changed. Even at the same ratio of effector cells and target cells (E : T), the distribution of the concentration of metabolites in individual cells presented a bimodal distribution. The correlation between metabolites and different apoptotic pathways in the early apoptosis cell group was established by the 4 metabolites at the single-cell level. Our results have revealed that the apoptosis of cancer cells induced by NK cells is a process with continuously changing metabolites. Even in the same cell line, the individual cancer cells still show different sensitivity to NK cells. Our work will provide new ideas for using metabolites to evaluate the toxicity of NK cells and reveal the process of apoptosis induced by NK cells in heterogeneous individual cancer cells.

## Results and discussion

### Acquisition of HepG2 single-cell metabolite profiles by CyESI-MS

**Fig. 1:**
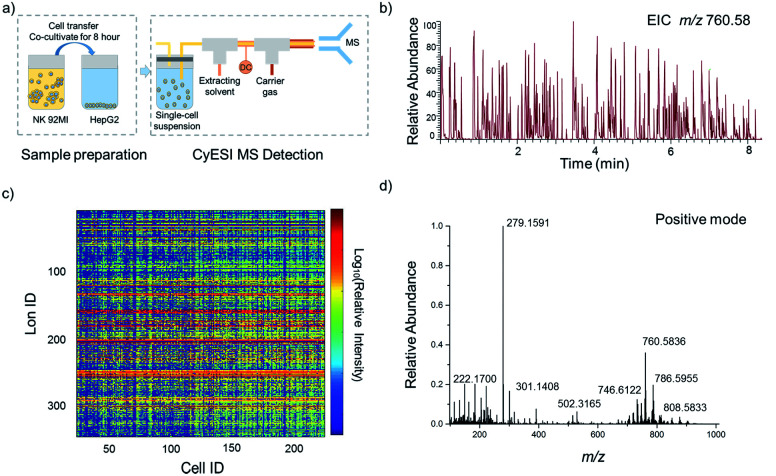
Acquisition of HepG2 single-cell metabolite profiles by CyESI-MS. (a) The procedure of single-cell sample preparation and CyESI-MS detection. (b) EIC of the cell-event marker ion at *m*/*z* 760.58 was extracted from the MS data in the positive-iron mode. The peak *m*/*z* 760.58 is a kind of abundant lipid PC 34 : 1 only existing in cells. (c) The heat map of 224 individual HepG2 cell MS profiles. (d) The normalized mass spectra of single HepG2 cells.

The experimental procedure is shown in Fig. 1a. HepG2 cells were used to estimate the metabolic profiling efficiency of CyESI-MS at first. The pulsed signals related to cell events were captured by MS (Fig. 1b). As shown in Fig. 1c, each column of the heat map represented a cell and each row represented a cell-related peak. We acquired 333 metabolic-related ions in each HepG2 cell. The normalized mass spectra of single HepG2 cells detected by CyESI-MS are shown in Fig. 1d. Because CyESI is considered as a soft ion source, the cell-related MS profile contains a variety of cellular small molecular metabolite information, such as protonated metabolite ions, which may be useful to reveal the process of apoptosis in cancer cells induced by NK cells.

### The dynamic changes of single-cell metabolic profiles after interaction with NK cells

**Fig. 2:**
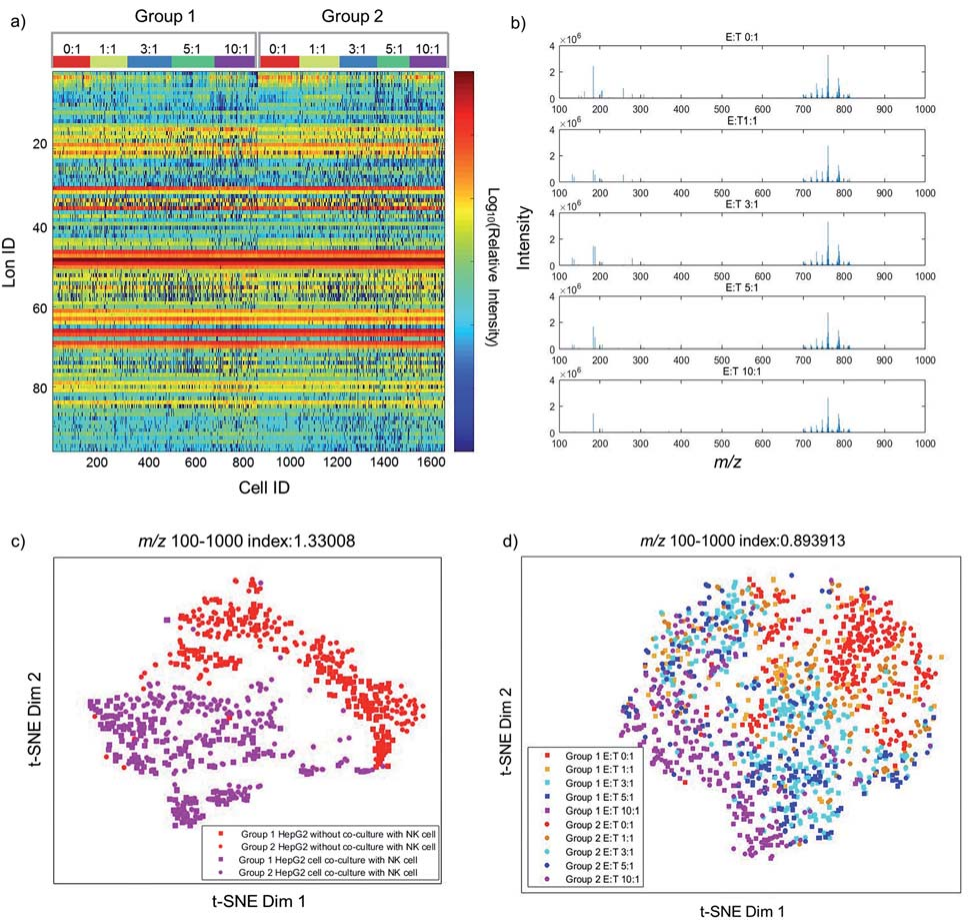
The dynamic changes of single-cell metabolic profiles after interaction with NK cells. (a) The heat map of two groups of single HepG2 cells with different ratios of E : T. In one group, 162, 102, 201, 195, and 200 cells were detected with the ratio of E : T from 0 : 1, 1 : 1, 3 : 1, and 5 : 1 to 10 : 1. In the other group 186, 160, 143, 115, and 179 cells were detected. (b) The normalized MS profiles of HepG2 cells with the ratio of E : T from 0 : 1 to 10 : 1. (c) The metabolic differences with and without co-culture with NK cells. (d) The consecutive distribution of HepG2 cells with different ratios of E : T from 0 : 1 to 10 : 1, from the single-cell metabolic profile analysis.

Previous studies have already elucidated the toxicity of NK cells to cancer cells, but few studies have reported the metabolite changes at the single-cell level. Therefore, we investigate the dynamic changes of metabolites in the process of cancer cell apoptosis induced by NK cells. The metabolic profiles of individual HepG2 cells co-cultured with different amounts of NK cells are shown in Fig. 2a. As shown in Fig. 2a, single-cell metabolic profiles are different even after co-culturing with the same amount of NK cells, which reveal the metabolic heterogeneity of HepG2 cells during apoptosis. The normalized MS profiles of HepG2 cells provide an overview of the distribution of metabolites after co-culturing with different amounts of NK cells (Fig. 2b). A cluster of peaks at *m*/*z* 700–1000 with high intensity including *m*/*z* 760.59, *m*/*z* 762.59, *m*/*z* 782.57, and *m*/*z* 808.58 are found with all ratios of E : T. The intensity of some small molecules at *m*/*z* 100–700 such as *m*/*z* 132.13, *m*/*z* 184.07, and *m*/*z* 308.08 reduces with the increasing ratio of E : T.

To visualize the differences of metabolic profiles, an unsupervised nonlinear learning algorithm named t-SNE was applied to cluster individual HepG2 cells by their metabolic fingerprints. After co-culturing with NK cells, HepG2 cells are clearly distinguished from those without co-culturing with NK cells (Fig. 2c). The results show that metabolic fingerprints can be used for distinguishing the normal cells and the apoptotic cells induced by NK cells. As shown in Fig. 2d, we found that HepG2 cells are located at the right side with a low ratio of E : T. With the increasing amount of NK cells, the distribution of HepG2 cells gradually moves from the right to the left. The distribution of HepG2 cells illustrates the consecutive dynamic changes of metabolites during apoptosis induced by NK cells.

### The changes of specific metabolites in HepG2 cells induced by NK cells

**Fig. 3:**
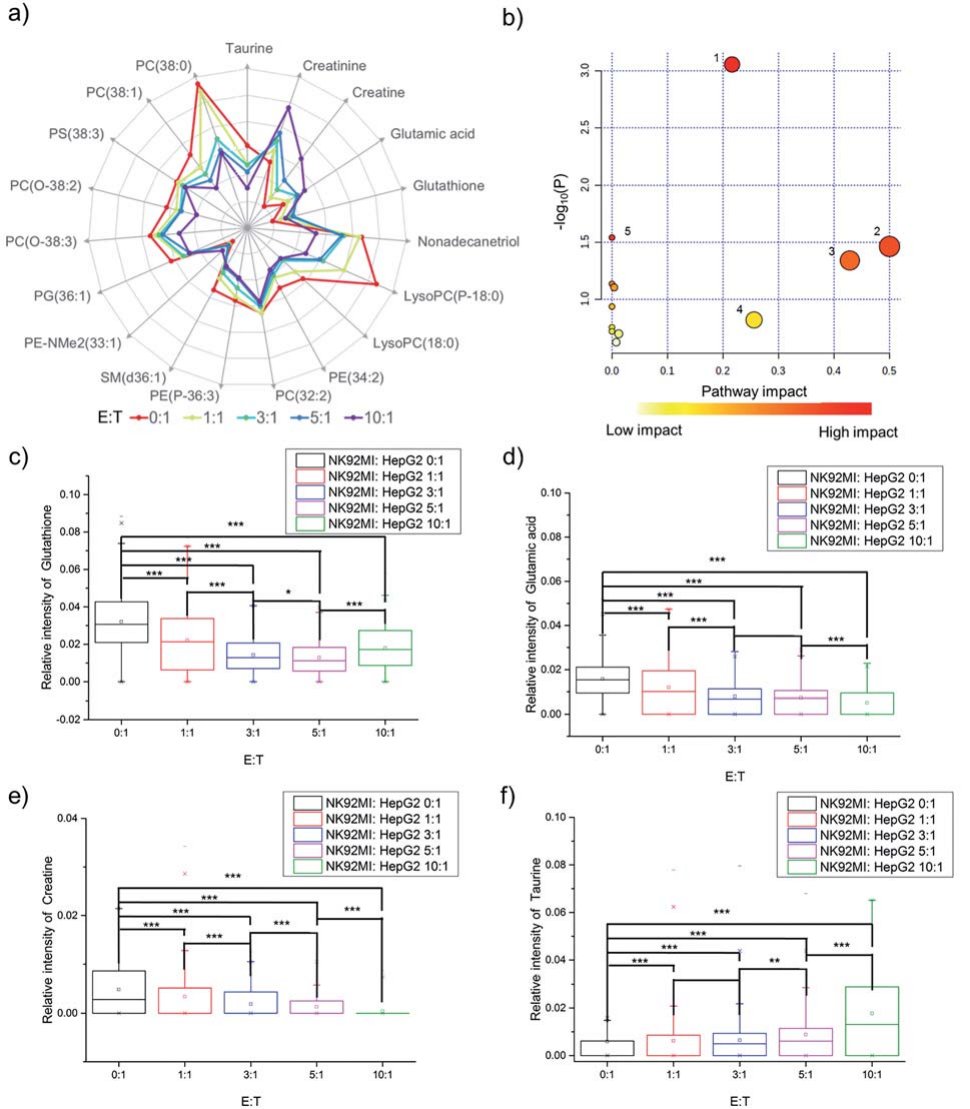
The changes of specific metabolites in HepG2 cells induced by NK cells. (a) Logarithmic radar charts of average relative intensities of 19 characteristic metabolites with different ratios of E : T. (b) Several metabolic pathways are impacted during cell apoptosis induced by NK cells: (1) glycerophospholipid metabolism, (2) d-glutamine and d-glutamate metabolism, (3) taurine and hypotaurine metabolism, (4) glutathione metabolism, and (5) glycine, serine and threonine metabolism. The changes of relative intensities of (c) glutathione, (d) glutamic acid, (e) creatine, and (f) taurine in individual cells during apoptosis and the *p*-value indicates a significant difference (using Bonferroni to adjust the *p*-value: *p < 0.1/4, **p < 0.05/4, and ***p < 0.005/4).

The fingerprints could only reveal the dynamic changes of metabolites by fuzzy recognition, but the accurate *m*/*z* could help to annotate the metabolites. We annotate the metabolites by accurate *m*/*z* and calculate the average relative intensity of single-cell metabolites. We find the relative intensities of 19 metabolites, including 7 small molecules at *m*/*z* 100–700 and 12 lipids at *m*/*z* 700–1000, continuously change with the increase of the number of NK cells (Fig. 3a, S1 and Table S1†). The 19 metabolites are involved in several metabolic pathways (Fig. 3b). The most significant impact occurs in the glycerophospholipid metabolic pathway which hits 3 kinds of metabolites including LysoPC, PC and PE. The impact of glycerophospholipid metabolism might be caused by membrane perforation on HepG2 cells which was induced by the PFP and granzyme released by NK cells. Multiple types of lipids are involved in the glycerophospholipid metabolic pathway, so it is difficult to establish a correlation with a specific lipid.

d-Glutamine and d-glutamate metabolism, taurine and hypotaurine metabolism, glutathione metabolism, and glycine, serine and threonine metabolism each hits one metabolite, including d-glutamic acid, taurine, glutathione and creatine. The changes in the above four pathways might be caused by intracellular lysosome rupture during cell apoptosis.

The metabolite changes may relate to cell apoptosis. It has been illustrated that the decrease of glutathione is a potential early activation signal to apoptosis, and the subsequent generation of reactive oxygen species (ROS) promotes cell apoptosis.^54,55^ With the increasing amount of NK cells, the relative intensity of glutathione decreases (Fig. 3c). The statistically significant difference not only exists between the groups with and without co-culturing with NK cells, but also exists between the groups co-cultured with different amounts of NK cells. Similar results could be obtained in glutamic acid and creatine (Fig. 3d and e). NK cells lead to perforation on cancer cells membrane, which will further result in the leakage of some metabolites during the cell apoptosis, including glutamic acid^56–58^ and creatine.^59,60^ The results demonstrated that the changes of specific metabolites could be used to reveal the process of cell apoptosis.

As reported in a previous study, the increasing concentration of taurine in cancer cells can help to fight against cell apoptosis.^61–63^ Similar results can be found in the cell apoptosis of HepG2 cells induced by NK cells. With the increasing amount of NK cells, the relative intensity of taurine increases (Fig. 3f).

### The metabolic heterogeneity of the response of HepG2 cells induced by NK cells

The average cell metabolic data reveal the continuous changes of specific metabolites during cell apoptosis, but they cover up the metabolic heterogeneity of the cells. The single-cell metabolite information is important especially for studying cell apoptosis, because each cell has a specific metabolite abundance in different apoptotic states. The distributions of single-cell specific metabolites could help to reveal the metabolic heterogeneity.

**Fig. 4:**
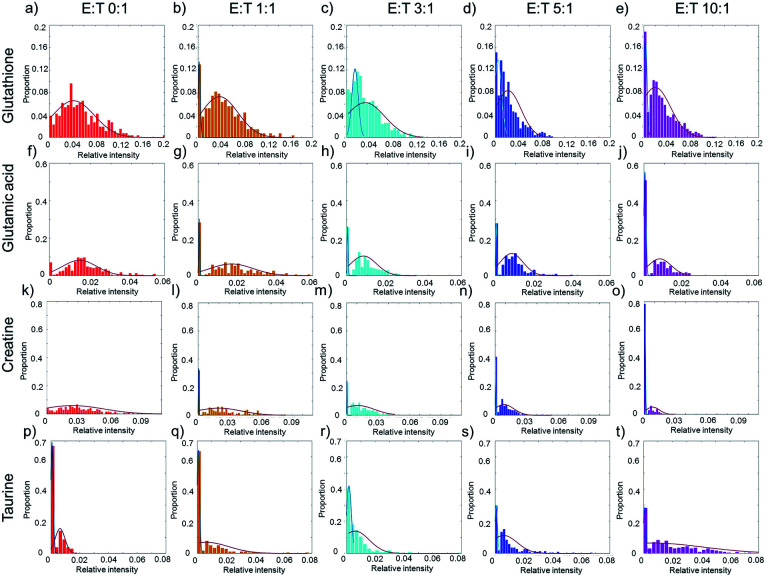
The heterogeneity of the response of HepG2 cells induced by NK cells. (a)–(e) The distribution of glutathione with different ratios of E : T. (f)–(j) The distribution of glutamic acid with different ratios of E : T. (k)–(o) The distribution of creatine with different ratios of E : T. (p)–(t) The distribution of taurine with different ratios of E : T. The red curves in (a), (f), and (k) show the metabolic heterogeneity in HepG2 cells without co-culturing with NK cells. In (b)–(e), (g)–(j), and (i)–(o), the cells are divided into two phenotypes according to the relative intensity of specific metabolites. The blue curves represent the cells of low relative intensity and may be in the stage of late apoptosis, and the red curves represent the cells of high relative intensity and may be in the stage of early apoptosis. In (p)–(t) the blue curves represent the cells in low relative intensity of taurine, while red curves represent the cells in high relative intensity.

Taking glutathione as an example, the relative intensity of glutathione in normal HepG2 cells disperses in a wide range without co-culturing with NK cells (Fig. 4a). The distribution of glutathione can be fit in a one peak Gaussian function which indicates the existences of metabolic heterogeneity in single cells. After co-culturing with NK cells, the distribution of glutathione changes from one peak to two peaks (Fig. 4b–e), which indicates that the HepG2 cells might be divided into two phenotypes according to the relative intensity of glutathione. A mixture Gaussian function is used to describe the distribution of glutathione (Fig. S2†). One group of cells with low relative intensity of glutathione may be late apoptotic cells, and the other group of cells with high relative intensity of glutathione may be early apoptosis cells. With the increasing amount of NK cells, the proportion of cells in late apoptosis increases (Fig. S3a†). The proportion of cells in early apoptosis and the mid-value of the concentration of glutathione decrease when more NK cells are added (Fig. S3e†). Similar results are obtained in glutamic acid and creatine (Fig. 4f–o, S3b, c, f and g†).

Taurine could be absorbed by cells to fight against adverse conditions. Without co-culturing with NK cells, only a small proportion of HepG2 cells have high relative intensity of taurine (Fig. 4p). With the increasing amount of NK cells, the proportion of HepG2 cells with high relative intensity of taurine and the mid-value of the concentration of taurine increase (Fig. 4q–t, S3d and h†). The results show that the metabolic heterogeneity can be used to distinguish the cell phenotypes and reveal the different apoptotic stages of each single cell.

### The correlation between metabolites and the apoptotic pathway and different apoptotic pathways in the early apoptosis group of HepG2 cells induced by NK cells

The activity of the apoptotic pathway can be inferred by measuring the quantity and activity of specific proteins. However, measuring the proteins at the single-cell level, especially at the same time in individual cells, is still a problem. Metabolites, as substrates for protein catalytic reactions, can also be used to infer the activity of the apoptotic pathway. The concentration of different metabolites in individual cells will represent the activity of the apoptotic pathway.^62,64,65^

**Fig. 5:**
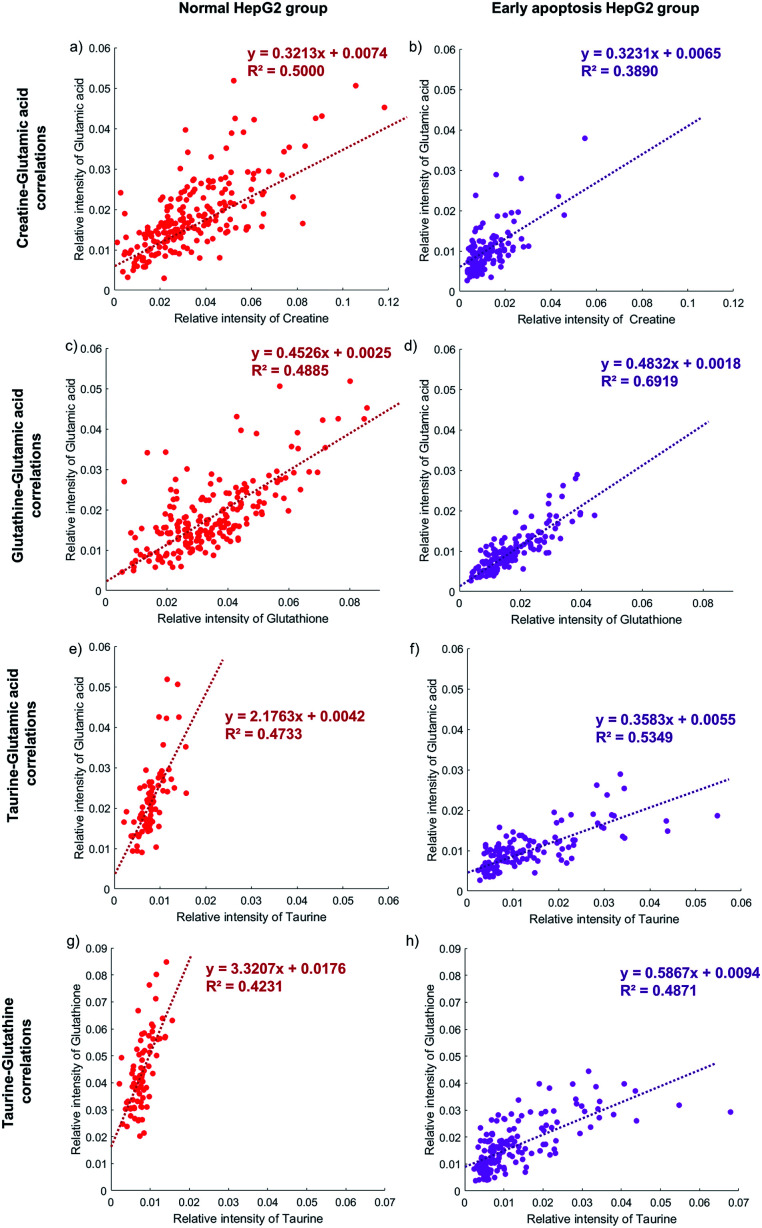
The correlation between four metabolites in the normal HepG2 group and early apoptosis HepG2 group. The normal HepG2 group is on the left represented by red dots and the early apoptosis HepG2 group is on the right represented by purple dots. (a) and (b) The glutamic acid is positively correlated with creatine in both groups with similar slopes, as well as glutamic acid and glutathione (c) and (d). (e) and (f) The glutamic acid is positively correlated with taurine in both groups but with different slopes, as well as glutamic acid and glutathione (g) and (h). 207, 124, 179, 154, 78, 135, 77, and 154 cells are obtained in each figure from (a) to (h).

The correlation between the concentration of metabolites and the activity of the apoptotic pathway is first proved in individual cells. Taking glutamic acid and creatine as an example, the relative intensity of glutamic acid and creatine is closely related to the membrane perforation during cell apoptosis and their changes should be consistent. The relative intensity of glutamic acid and creatine in individual normal HepG2 cells is shown in Fig. 5a. The concentration of glutamic acid is positively correlated with the concentration of creatine in normal HepG2 cells. Similar results can be found in early apoptosis cells induced by NK cells. The slopes of the early apoptosis HepG2 group and normal HepG2 group are similar, which illustrates that the concentration of metabolites can represent the activity of the apoptotic pathway. The concentration of both metabolites is lower in the early apoptosis HepG2 group (Fig. 5b), which indicates the high activity of the membrane perforation apoptotic pathway.

The correlation between different apoptotic pathways can be established by specific metabolites. The concentration of glutamic acid represents the activity of membrane perforation and the concentration of glutathione represents the activity of the ROS apoptotic pathway. In the normal HepG2 group, the concentration of glutamic acid is positively correlated with the concentration of glutathione (Fig. 5c), and in the early apoptosis HepG2 group (Fig. 5d). The slopes in both groups are similar, showing that the process of membrane perforation and ROS apoptotic pathway are consistent. With the lower concentration of glutamic acid and glutathione, the individual cells in the early apoptosis HepG2 group show high activity of membrane perforation and ROS apoptotic pathway.

Specific metabolites can also affect the activity of the apoptotic pathway. The concentration of taurine is positively correlated with the concentration of glutamic acid in the early apoptosis HepG2 group (Fig. 5f), but the concentration of glutamic acid of most individual cells is still lower than that in the normal HepG2 group (Fig. 5e), which indicates that the intake of taurine can help cells to resist apoptosis, but can't completely counteract the effects of membrane perforation apoptosis induced by NK cells. Similar results can be found in the ROS apoptotic pathway (Fig. 5g and h).

## Conclusions

In this work, we used the dynamic metabolite changes of individual HepG2 cells to reveal the process of apoptosis induced by NK92MI cells through label-free CyESI-MS. A large amount of chemical information from single HepG2 cells was obtained which were unreachable by conventional FCM or CyTOF without using antibodies. Metabolic fingerprints were used to cluster the HepG2 cells and illustrate the consecutive dynamic changes of metabolites at the single-cell level. The specific metabolite changes could help to reveal the process of apoptosis. The concentration of specific metabolites can be used to not only distinguish cell phenotypes during the apoptosis, but also establish the correlation between different apoptotic pathways in the early apoptosis group. This work provided a new thought for using single-cell specific metabolites to reveal the metabolic heterogeneity in cell apoptosis which would be a powerful means for evaluating the cytotoxicity of NK cells and studying the apoptotic pathway at the single-cell level.

## Data availability

All experimental data, procedures for data analysis are provided in the ESI.†

## Author contributions

Z. S. designed and optimized experiments. H. Z developed the data analysis methods. H. Y. maintained the Cy-ESI device. X. P. cultured the cells. J. Y. modified the figures. S. Z., G. H. and X. Z. revised the manuscript provided by the other authors. All the authors discussed the results and contributed to edit the manuscript.

## Conflicts of interest

There are no conflicts of interest to declare.

## Supplementary Material

SC-013-D1SC06366A-s001
