# Supplementary material for: Dynamic metabolic change of cancer cells induced by natural killer cells at the single-cell level studied by label-free mass cytometry
Source: Chem Sci. 2022 Jan 3;13(6):1641–7. doi: 10.1039/d1sc06366a (PMC8827047; doi:10.1039/d1sc06366a)
Supplement: SC-013-D1SC06366A-s001 [file SC-013-D1SC06366A-s001.pdf]

Electronic Supplementary Material (ESI) for Chemical Science.
This journal is © The Royal Society of Chemistry 2022

Supplementary Information for:

Dynamic metabolic change of cancer cells induced by natural killer cells at single-cell level studied by label-free mass cytometry

Zizheng Shen,[a] Hansen Zhao, [a] Huan Yao, [a] Xingyu Pan, [a] Jinlei Yang, [a] Sichun Zhang,*[a] Guojun Han*[bcd] and Xinrong Zhang[a]

[a] Department of Chemistry, Tsinghua University, Beijing 100084, P.R. China.
[b] Institute of Medical Technology, Peking University Health Science Center, Beijing, 100191, P.R. China.
[c] Peking University School and Hospital of Stomatology, Beijing, 100081, P.R. China.
[d] Department of Biomedical Engineering, Peking University Health Science Center, Beijing, 100191, P.R. China.

*Prof. Sichun Zhang: sczhang@mail.tsinghua.edu.cn

Table of Contents

Abbreviations..3

1. Experimental Section...4

1.1 *General chemistry information*...4

1.2 *Cell culture and handling*..4

1.3 *CyESI Configuration*...4

1.4 *CyESI-MS analysis*..5

1.5 *Solvent optimization*..5

1.6 *Individual cell data extraction and background elimination*..................5

1.7 *Statistical analysis*...6

1.8 *Metabolites annotation and pathway analysis*..6

Figure S1. The hot map of 19 specific metabolites in individual cell..................7

Figure S2. The R^2 of using different number of mixture Gaussian functions.......8

Figure S3. The dynamic distribution of four specific metabolites.......................9

Figure S4. The calculation of NK92 MI cells and HepG2 cells. 10

Figure S5. The pictures of co-culturing NK92 MI with HepG2. 11

Figure S6. The differences between NK 92MI and HepG2 under microscope. .12

Table S1. 19 metabolites with continuously changes in different ratio of E:T. ..13

Abbreviations

CyESI-MS	Coupling Flow Cytometry to ESI-MS
DPBS	Dulbecco's Phosphate Buffered Saline
E:T	Ratio of Effector cells and Target cells
ESI	Electrospray Ionization
FCM	Flow Cytometry
GSH	Glutathione
HepG2	Human Hepatocellular Carcinoma
HPLC	High performance liquid chromatography
LDH	Lactate Dehydrogenase
MS	Mass Spectrometry
NK cell	Natural Killer cell
PFP	Pore-forming Protein
TOF	Time of Flight

1. Experimental Section

1.1 *General chemistry information*

The HPLC-grade methanol and MS-grade (≥99%) ammonium formate were purchased from Sigma-Aldrich (St. Louis, MO, USA). Inositol,β-mercaptoethanol, folic acid were purchased from Aladdin Chemicals (Shanghai, China). The gas including CO_2 and N_2 were purchased from Liquefied Air Co., Ltd., (Tianjin, China).Ultrapure water (re-sistance ≥18 $M\Omega \cdot cm^{-1}$) was prepared in laboratory used Milli-Q water purification system. All the cell cultured reagents including MEM α, MEM, HS, FBS, P/S, trypsin, DPBS were all purchased from Gibco Life Technologies (Carlsbad, CA, USA).

1.2 *Cell culture and handling*

NK92 MI and HepG2 were used in the experiment. NK92 MI cell line was separated from peripheral blood mononuclear cells of a man with rapidly progressive non-Hodgkin's lymphoma and was modified by transfection to make it an IL-2-independent NK cell line. HepG2 cell line was a human liver carcinoma cell line, derived from a well-differentiated hepatocellular carcinoma. Both cell lines were purchased from Shanghai Enzyme Research Biotechnology Co. Ltd. (Shanghai, China). NK92 MI cells were cultured in MEM α with 0.2 mM inositol, 0.1 mM β-mercaptoethanol, 0.02 mM folic acid, 12.5% horse serum (HS), 12.5% fetal bovine serum (FBS), 1% penicillin-streptomycin (P/S) (100 U/mL). HepG2 were cultured in MEM with10% FBS, 1% P/S (100 U/mL). Both cell lines were cultural at 37°C and 5% of CO_2.

NK92 MI cells and HepG2 cells are calculated by Flow Cytometry (figure S4) and cultured together in different ratio of E:T from 0:1, 1:1, 3:1, 5:1, to 10:1 (figure S5) for eight hours. The mixed cells were cultured in the NK92 MI medium mentioned above. Because the NK92 MI cells were suspension cells and HepG2 cells were adherent cells, so they were separated by removing the medium (figure S6). While removing the cell culture medium, we also removed the NK cells suspended in it. The HepG2 cells were remained at bottom of the flask. Only HepG2 cells were used for further analysis.

For further CyESI-MS analysis, HepG2 cells were washed by Dulbecco's Phosphate Buffered Saline and digested with trypsin in short time (less than 30 seconds). The cells were re-suspended with Dulbecco's Phosphate Buffered Saline and spun down at 1000 rpm for 5 min to remove the cell debris. 5×10^4 HepG2 cells were collected and finally re-suspended into 1 mL 140 mM ammonium formate aqueous solution (pH = 7.4). The final cell concentration was 5×10^4 cells/mL.

1.3 *CyESI Configuration*

The hand-made CyESI system was composed of three parts: cell sampling, online extraction, and electrospray ionization. Cell sampling part included a glass bottle and a syringe which was used to inject gas to bottle. The dispersed cells were put in the bottle and was pumped in to capillary by air pressure. Online extraction part was composed by three coaxial silicon capillaries, which is 150 μm O.D., 50 μm I.D., 150 μm O.D., 50 μm I.D., and 750 μm O.D., 530 μm I.D.. Briefly speaking, the inner capillary was used to introduce the cell suspension, the middle capillary was used to transport the extracting solvent, and the outer capillary was used to transfer the carrier gas. The distance between the end of inner capillary and the end of middle capillary was adjust into 1.5 mm. In electrospray ionization part, high voltage direct current was added. The details of the device can be seen in the previous work.

1.4 *CyESI-MS analysis*

The CyESI-MS platform consisted of a CyESI ion source and a QE-Orbitrap mass spectrometer (Thermo Scientific, San Jose, CA), separating around 5 mm. The HepG2 cells suspension (5×10^4 cells/mL) was introduced into the inner capillary at the flow rate of 1 μL/min (50 cells/min). The flow rate of the extracting solvent in the middle capillary was set to 10 μL/min. Nitrogen was chosen as the carrier gas and the pressure was around 0.5 MPa. The high positive DC voltages of 2.3 KV were applied to the extracting solvent. Parameters of MS analysis were set as follows: the temperature of capillary was set at 320°C, no sheath gas, aux gas and sweep gas, the mass range was set at *m/z* 100-1000, the mass accuracy is 5 ppm, the resolution was set at 35000, AGC target was set at 10^6, maximum inject time was set at 50 ms, and microscan was set at 1. Mass calibration was needed before every experiment in order to get the accurate *m/z*.

1.5 *Solvent optimization*

The species of the extracting solvent was optimized to improve the detection coverage and the signal-to-noise ratio. With the growing ratio of methanol to H_2O, both detection coverage and the signal-to-noise ratio increased. Therefore, the 100% methanol was selected as the extracting solvent.

1.6 *Individual cell data extraction and background elimination*

The aim of individual cell data extraction to change the raw data into single-cell metabolic data. Firstly, the raw encrypted MS data files were transformed into accessible data through our group self-made program based on Matlab 7.0 (Mathworks, Natick, MA). Secondly, the pulse peaks *m/z* 760.58 (a kind of abundance lipid PC 34:1 only exists in cells) was used as the marker of the appearance of cell events, so that the consecutive complicated mass spectra was cut into slices witch each spectra may represent a cell event. Next, only the spectra with the signal-to-noise ratio of marker above 3 was saved as detectable cell-related signals. Then, lipid PC (34:1) and

intercellular metabolite GSH were used as gate to eliminate the multi-cell events and the fragment of the cell with the signal intensity significantly larger and smaller than individual cell events. Finally, the individual cell events were identified and cell data was extracted. After extracting the individual cell data, the background ions were subtracted from the individual cell data by removing all ions present in the extraction solvent and background.

1.7 *Statistical analysis*

We used an unsupervised analysis t-distributed stochastic neighbor embedding (t-SNE) to analysis the changes of metabolites and cluster the individual cell between different ratio of E:T. The average relative intensity of metabolites was calculated through adding the relative intensity of metabolites in each individual cell together and divided by the number of cells. The metabolites, whose average relative intensity continuously increased or decreased when adding more NK cells, were selected and concluded as specific metabolites. Two independent samples t-test was used to determine whether there were statistically significant differences between two groups, because the cell events in different ratio of E:T were not same. We also used Bonferroni to adjust our p-value to improve the reliability. Gaussian function and mixture Gaussian function were used to describe the distribution of the concentration of the specific metabolites in individual cell of different ratio of E:T. Linear fitting was used to verify and establish the correlation between the concentration of metabolites and the activity of the apoptotic pathway.

1.8 *Metabolites annotation and pathway analysis*

We used accurate mass to annotate the metabolites by matching with the standards mass in the Human Metabolome Database (http://www.hmdb.ca/). The metabolites could be annotated only if the difference between the accurate mass of metabolites and the standards mass of $(M+H)^+$ or $(M+Na)^+$ of the metabolites in database was less than 10 ppm. The metabolic pathways were analyzed using MetaboAnalyst 4.0 (http://www.metaboanalyst.ca/).

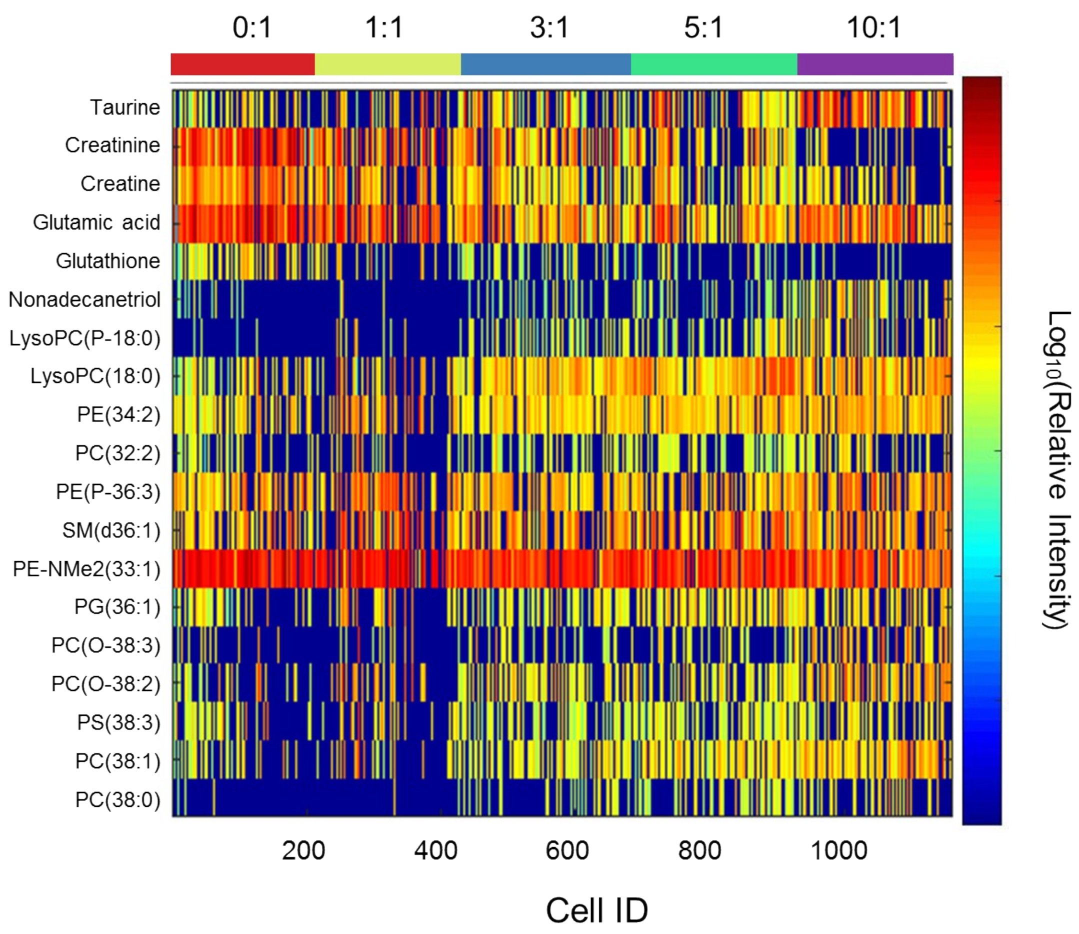

Figure S1. The hot map of 19 specific metabolites in individual cell.

The distribution of 19 specific metabolites among different ratio of E:T from 0:1 to 10:1 in individual HepG2 cell. Each line represents a metabolite and each row represents an individual cell.

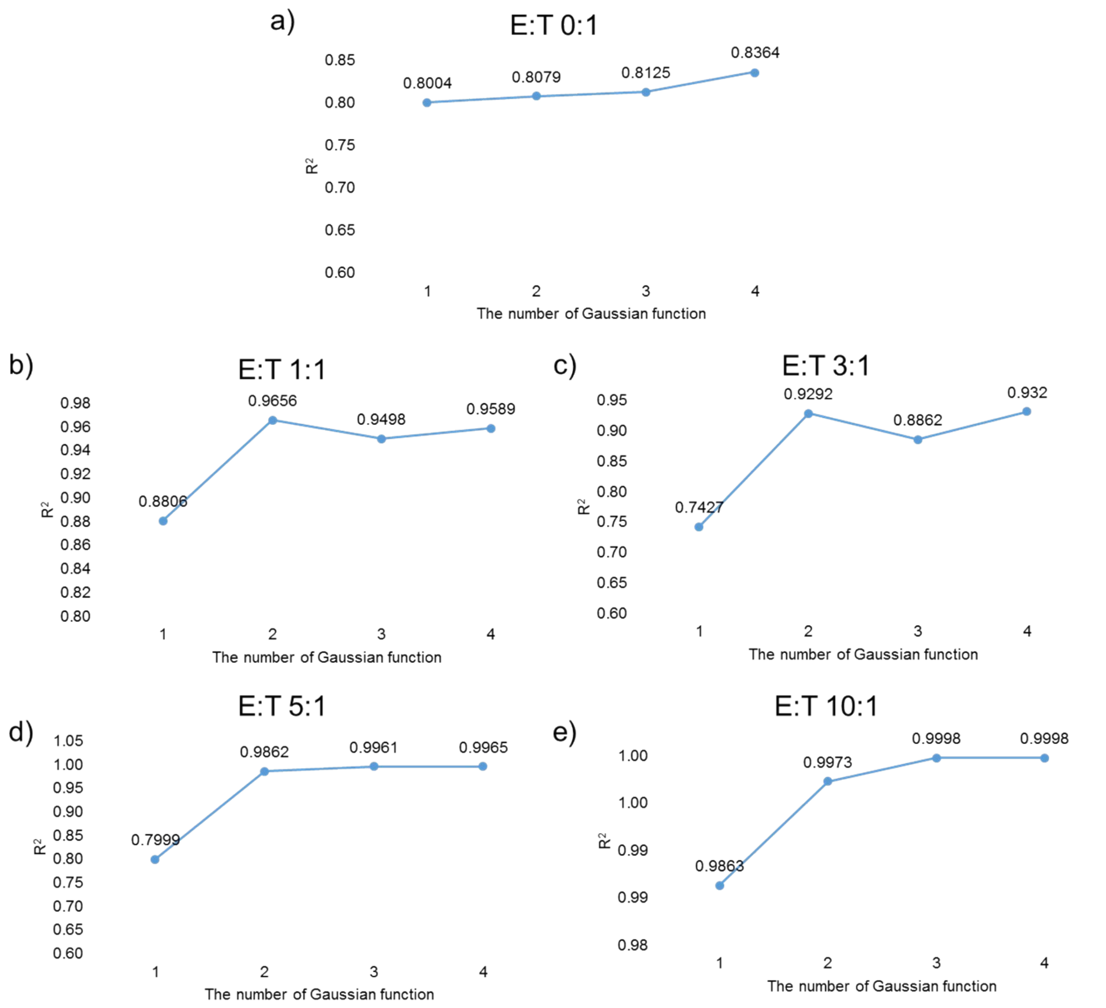

Figure S2. The R^2 of using different number of mixture Gaussian functions.

Take GSH as an example: a) In E:T 0:1, there was no turning point, so one Gaussian function is suitable. b)-e), in E:T 1:1 to 10:1, there was a turning point at two Gaussian functions, so we choose two Gaussian functions to fit the distribution of GSH. The same means were used to determine the number of mixture Gaussian functions in creatine, glutamic acid and taurine.

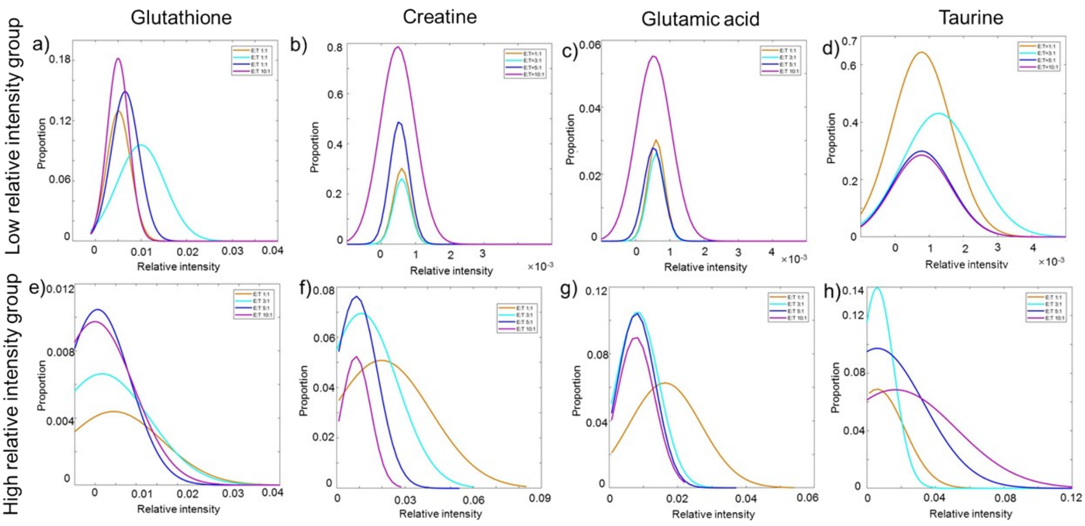

Figure S3. The dynamic distribution of four specific metabolites.

The cells can be divided in to two groups: one is low relative intensity group and the other is high relative intensity group The integral of the curve represents the proportion of the cells in a certain phenotype and the mid-value of the curve represents the average metabolite abundance of the cells in a certain phenotype. a)-c) With the increase amount of NK cells, the proportion of cells in low relative intensity group increases. e)-g) With the increase amount of NK cells, the proportion of cells in high relative intensity group decreases, and the mid-value of relative intensity of specific metabolites decrease. d) h)With the increase amount of NK cells, not only the proportion of HepG2 cells in high relative intensity of taurine increases, but also the mid-value of relative intensity of taurine increases.

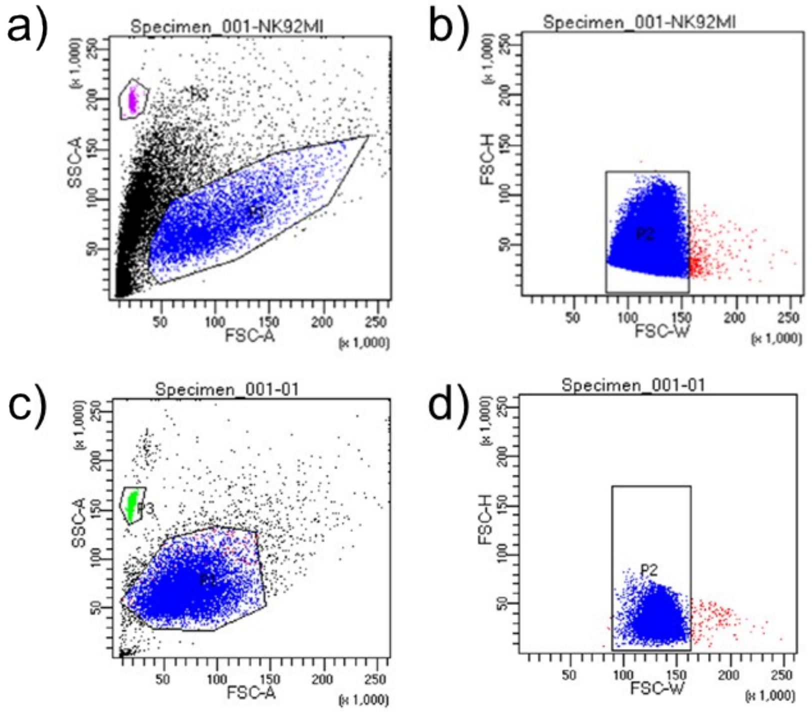

Figure S4. The calculation of NK92 MI cells and HepG2 cells.

The exact number of NK92 MI cells and HepG2 cells were calculate by absolute counting beads. The purple dots in a) and the green dots in c) were the absolute counting beads. The blue dots in b) were the single NK92 MI cells and in d) were the single HepG2 cells.

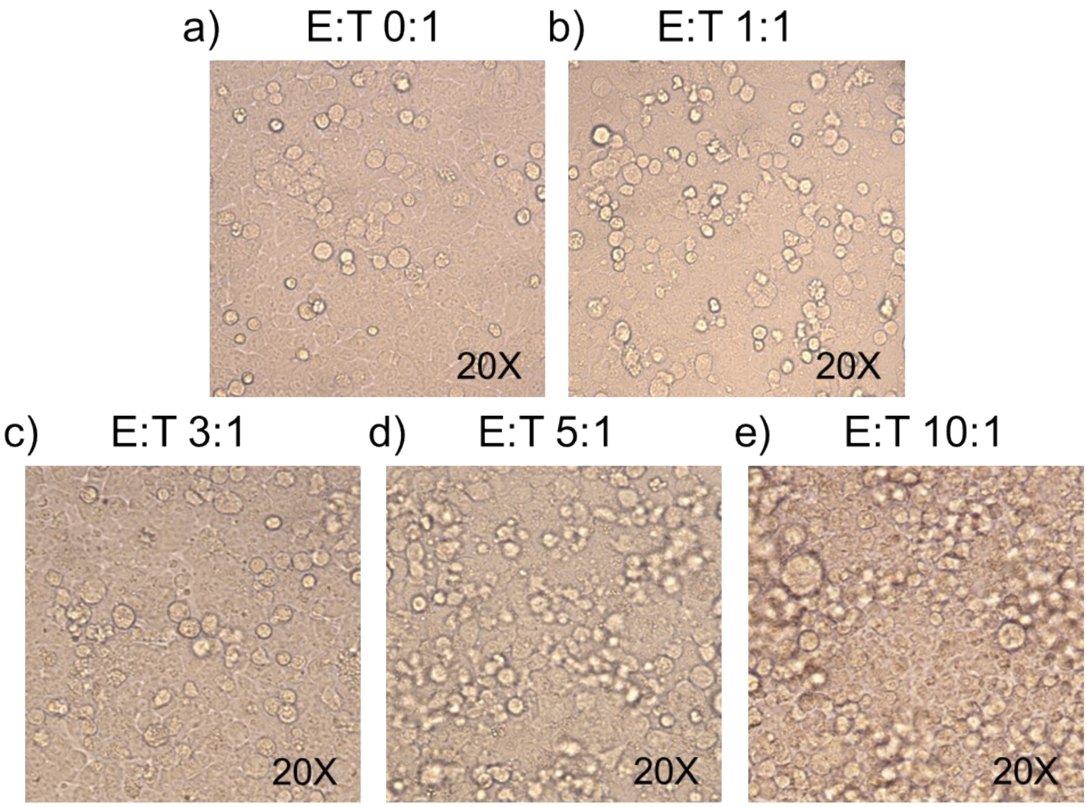

Figure S5. The pictures of co-culturing NK92 MI with HepG2.

NK92 MI cells and HepG2 cells were cultured together in different E:T from 0:1, 1:1, 3:1, 5:1, to 10:1 for 8 hours. With the increasing of E:T, the edge of HepG2 cells became blurred after co-culturing with NK 92MI cells.

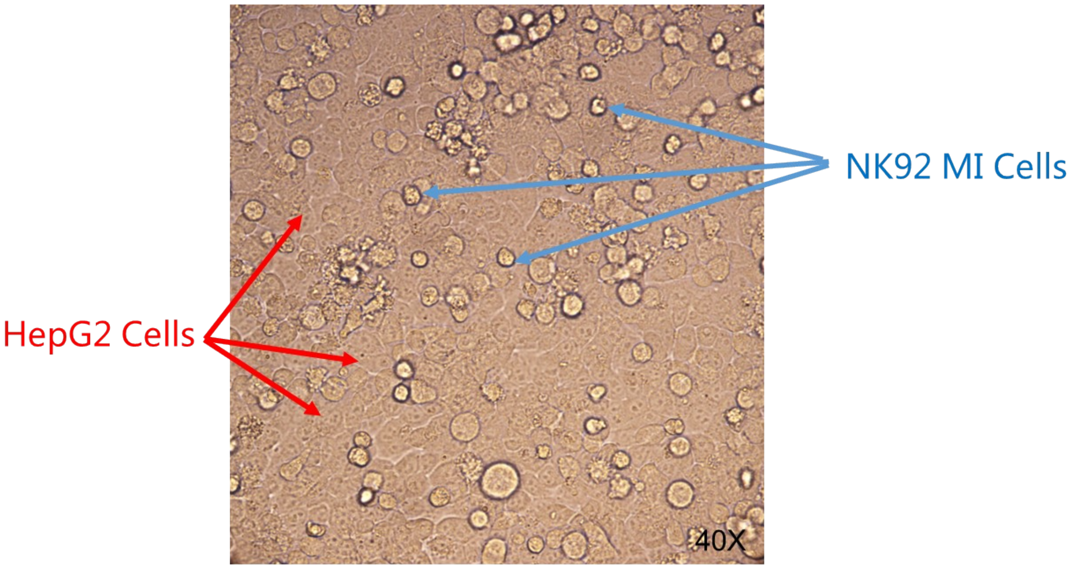

Figure S6. The differences between NK 92MI and HepG2 under microscope.

NK 92MI cells could be separated from HepG2 cells directly by removing the medium using peptide. NK92 MI cells (pointed by the red arrow) were suspension cells and HepG2 cells were anchorage-dependent cell (pointed by the blue arrow), so they were easily separated by peptide.

Table S1. 19 metabolites with continuously changes in different ratio of E:T.

The metabolites were annotated by accurate *m/z* in the positive ion mode (scan range *m/z* 100-1000). The difference between the accurate mass of metabolites and the standards mass of $(M+H)^+$ or $(M+Na)^+$ of the metabolites in database was less than 5 ppm. * means there may be other types of lipids.

No.	Observed mass, *m/z*	Compound name	Formula	Adduct	Theoretical mass, *m/z*	Error, ppm
1	126.0220	Taurine	$C_2H_7NO_3S$	M+H	126.0219	0.79
2	136.0481	Creatinine	$C_4H_7N_3O$	M+Na	136.0481	0.00
3	154.0586	Creatine	$C_4H_9N_3O_2$	M+Na	154.0587	0.65
4	170.0423	Glutamic acid	$C_5H_9NO_4$	M+Na	170.0424	0.59
5	330.0725	Glutathione	$C_{10}H_{17}N_3O_6S$	M+Na	330.0730	1.51
6	339.2884	Nonadecanetriol	$C_{19}H_{40}O_3$	M+Na	339.2870	4.13
7	508.377	LysoPC(P-18:0)*	$C_{26}H_{54}NO_6P$	M+H	508.3760	1.97
8	524.3706	LysoPC(18:0) *	$C_{26}H_{54}NO_7P$	M+H	524.3711	0.95
9	700.5271	PE(34:2) *	$C_{39}H_{74}NO_7P$	M+H	700.5276	0.71
10	714.5427	PC(32:2) *	$C_{40}H_{76}NO_7P$	M+H	714.5432	0.70
11	726.5438	PE(P-36:3) *	$C_{41}H_{76}NO_7P$	M+H	726.5432	0.83
12	731.6027	SM(d36:1) *	$C_{41}H_{83}N_2O_6P$	M+H	731.6062	4.78
13	754.5321	PE-NMe2(33:1) *	$C_{40}H_{78}NO_8P$	M+Na	754.5357	4.77
14	777.5618	PG(36:1) *	$C_{42}H_{81}O_{10}P$	M+H	777.5640	2.83
15	798.6402	PC(O-38:3) *	$C_{46}H_{88}NO_7P$	M+H	798.6371	3.88
16	822.6323	PC(O-38:2) *	$C_{46}H_{90}NO_7P$	M+Na	822.6347	2.92
17	836.5391	PS(38:3) *	$C_{44}H_{80}NO_{10}P$	M+Na	836.5412	2.51
18	838.6264	PC(38:1) *	$C_{46}H_{90}NO_8P$	M+Na	838.6296	3.82
19	840.6429	PC(38:0) *	$C_{46}H_{92}NO_8P$	M+Na	840.6453	2.85
